# A Narrative Review of Ultrasound-Guided and Landmark-based Percutaneous Cryoneurolysis for the Management of Acute and Chronic Pain

**DOI:** 10.1007/s11916-024-01281-z

**Published:** 2024-07-04

**Authors:** Rodney A. Gabriel, Eri C. Seng, Brian P. Curran, Paul Winston, Andrea M. Trescot, Igor Filipovski

**Affiliations:** 1 University of California, San Diego, La Jolla, CA California, USA; 2 University of British Columbia, Vancouver, Canada; 3 Florida Pain Relief Group, Tampa, FL USA; 4 Copenhagen Cryo Center, Copenhagen, DNK Denmark

**Keywords:** Cryoneurolysis, Cryoanalgesia, Ultrasound, Acute pain, Chronic pain

## Abstract

**Purpose of Review:**

Cryoneurolysis refers to the process of reversibly ablating peripheral nerves with extremely cold temperatures to provide analgesia for weeks to months. With ultrasound-guidance or landmark-based techniques, it is an effective modality for managing both acute and chronic pain. In this review, we summarize the reported literature behind its potential applications and efficacy.

**Recent Findings:**

Here, we summarize several studies (from case reports to clinical trials) describing the use of ultrasound-guided and landmark-based cryoneurolysis for acute and chronic pain. Acute pain indications included pain related to knee arthroplasty, limb amputations, mastectomies, shoulder surgery, rib fractures, and burn. Chronic pain indications included chronic knee pain (due to osteoarthritis), shoulder pain, painful neuropathies, postmastectomy pain syndrome, phantom limb pain, facial pain/headaches, foot/ankle pain, inguinal pain, and sacroiliac joint pain.

**Summary:**

For both acute and chronic pain indications, more high quality randomized controlled clinical trials are needed to definitively assess the efficacy of cryoneurolysis versus other standard therapies for a multitude of pain conditions.

## Introduction

Cryoneurolysis (often termed cryoanalgesia when describing the analgesic effects of cryoneurolysis) refers to the process of reversibly ablating peripheral nerves with extremely cold temperatures to provide analgesia for weeks to months. It is an effective modality for managing both acute and chronic pain [1–3]. Although it was first described in 1961 as a use of liquid nitrogen to ablate nerves at temperatures of -190 °C, it took another 15 years before Lloyd and colleagues introduced the term “cryoanalgesia” when describing this method for pain control. There have been several modes of delivery described, particularly via open surgical approaches or percutaneous approaches using landmark or image-guided techniques, including computed tomography, magnetic resonance imaging, fluoroscopy, and ultrasound [3, 4].

Analgesia from cryoneurolysis results from the Wallerian degeneration that occurs distally to the treatment site. As the endoneurium, perineurium and epineurium remain unaffected at temperatures above − 100 °C, the nerve is able to regenerate at a rate of 1–2 mm per day. Once the nerve regenerates and conduction is restored, the block is resolved. Various gases have been used to produce the ice ball lesions for cryoanalgesia, including nitrous oxide, carbon dioxide, and argon. Temperatures colder than − 100 °C are associated with neural and stromal damage, can cause irreversible nerve injury, and may lead to neuroma formation [3]. The modern cryoprobe is comprised of a hollow outer tube, containing a smaller inner tube, that ends in a fine tip. Highly pressurized gas is ejected from the outer tube and allowed to expand in the closed probe tip. As the gas expands, the pressure drops decreasing the temperature to approximately − 70 °C (for nitrous oxide) and creating an ice ball at the tip, due to the Joule-Thomson effect [3]. The now lower pressure gas then flows back proximally through the inner tube to the console, where it is then vented. As the probe is sealed, gas is unable to escape and enter the patient’s tissues.

In this review, we gathered international experts - who have applied cryoneurolysis for acute and chronic pain in their daily clinical practices - to summarize the evidence behind its potential applications and efficacy. There are a limited number of clinical trials [5–13], thus, we also discuss results from observational studies [14–22] and case reports/series [23–46]. Furthermore, we focus on the application of cryoneurolysis of peripheral nerves using ultrasound and/or landmark techniques, rather than fluoroscopic, CT-guided, or open surgical approaches.

## Human and Animal Rights

All reported studies/experiments with human or animal subjects performed by the authors have been previously published and complied with all applicable ethical standards (including the Helsinki declaration and its amendments, institutional/national research committee standards, and international/national/institutional guidelines).

### Potential Risks

There are relatively few theoretical relative contraindications to cryoanalgesia, namely Reynaud syndrome, cryoglobulinemia and cold urticaria [3]. Additionally, the prolonged duration of sensory, motor, and proprioception block lasting weeks to months may not be appropriate for acute or chronic pain management. There has been reported a rare risk of myonecrosis [47]. The risks of cryoanalgesia are similar to other needle-based percutaneous procedures and include bleeding, bruising and infection. Specific risks include permanent injury to the nerve, injury to the surrounding tissue, and discoloration of the skin if the cannula is retracted prior to resolving the ice ball and allowed to contact other areas near the target site [3]. To mitigate these risks, cannulas designed specifically for superficial nerves should be used along with heating units at and below the skin.

There have been no published cases of permanent nerve injury, development of neuroma, or long-term changes to nerve function due to cryoanalgesia [3]. One study, however, noted that two patients (1.2%) reported severe dysesthesia in the area of treatment that interfered with sleep and daily activities [16]. Additionally, there have been two randomized, controlled clinical trials that reported an increase in neuropathic pain following cryoanalgesia during a thoracotomy [48, 49]. One study compared the efficacy of epidural infusion alone versus an epidural infusion with intercoastal cryoanalgesia [48]. The authors noted a higher incidence of neuropathic pain at 8 weeks that ultimately resolved by 6 months. The cryoanalgesia study group also reported higher pain scores at 12 h, 2 days, and 8 weeks. However, the statistical significance was bit adjusted for multiple comparisons and the results remain inconclusive. The second study analyzed the efficacy of epidural infusion alone versus intercostal cryoanalgesia alone [49]. Although the authors reported increased incidence of allodynia at 6 and 12 months in the patients who received cryoanalgesia, the study was not adjusted for multiple comparisons and, thus, also remained inconclusive.

In both clinical trials that reported allodynia, cryoanalgesia was administered via surgical exposure with possible nerve retraction. There is preclinical evidence that manipulating a nerve during cryoanalgesia may increase the risk of developing a chronic pain condition [50]. One hypothesis is that physically manipulating a nerve produces an afferent barrage resulting in central sensitization at the time of cryoanalgesia. The later axonal regeneration is then perceived as dysesthesia [51]. It is important to note, however, that there have been numerous other reports that have not identified an increased risk of this complication in both thoracotomies and other surgeries that involve cryoanalgesia [52]. Another possible explanation for these complications is that partial ablations could result in hyperalgesia, which could account for both the reports of severe dysesthesia and allodynia [50].

### Applications for Acute Pain Management

Cryoneurolysis is a potential option for various indications associated with acute pain related to surgery and trauma. Ultrasound-guided and landmark-based percutaneous cryoneurolysis has been described for various surgical populations, including knee arthroplasty [12–16, 45], limb amputations [40, 46], mastectomies [11, 41], and shoulder surgery [43]. For trauma indications, there are reports related to patients with rib fractures [38, 39, 44] and burns [10, 36].

Traditional approaches for extended acute analgesia (1 to ~ 7 days) for surgical patients include continuous perineural, fascial plane or neuraxial blocks [53]. For more than two decades, there have been several reports describing the benefits of continuous local anesthetic-based blocks [53, 54]; however, they are not without their limitations (some of which may be avoided with croyneurolysis). Important limitations of continuous blocks include: (1) limited duration of analgesia based on length at which catheters may remain in situ (dependent on available medication volume or risk of infection); (2) need for infusion pump and reservoir bag, which may be a burden to the patient; and (3) risk of catheter dislodgement, which could unexpectedly decrease the duration of analgesia. These limitations are addressed with cryoneurolysis as it may provide weeks to months of analgesia and is applied via a single administration. However, there are important points regarding cryoneurolysis that must be taken into account when considering its application for acute pain. These issues will depend on the target nerve in question. For example, prolonged motor and sensory blockade may not be desirable. Thus, careful and thoughtful consideration is warranted prior to applying cryoneurolysis for acute pain related to surgery.

#### Knee Arthroplasty

Two common target nerves for perioperative ultrasound-guided or landmark-based cryoneurolysis for knee surgery are the infrapatellar branch of the saphenous nerve (IBSN) and the anterior femoral cutaneous nerve (AFCN) [12, 14, 15, 43, 45]. These nerves both provide sensory innervation to the anterior aspect of the knee, and are reliable targets given their predictable and easily accessible locations. Specifically, the infrapatellar branch of the saphenous nerve and the anterior femoral cutaneous nerve innervate the inferior and superior aspects of the knee, respectively. Its potential analgesic effects have been described in some case reports [43, 45]. Outcomes following a preoperative blind approach to the infrapatellar branch of the saphenous nerve and anterior femoral cutaneous nerve were described in a retrospective study among 100 patients who underwent TKA [15]. Here, the patients who received cryoanalgesia had reduced incidence of prolonged hospitalization and decreased opioid consumption up to 12 weeks following TKA. Subsequently, two prospective studies have been reported [12, 13]. In a single center unblinded randomized controlled trial of 124 subjects, landmark-based cryoneurolysis of the infrapatellar branch of the saphenous nerve and anterior femoral cutaneous nerve did not reduce 6-week opioid consumption in the intention-to-treat analysis (but did in the per protocol analysis) [13].

Pairing the percutaneous cryoprobe with an ultrasound device may allow more specific targeting of the IBSN versus landmark techniques. In a retrospective analysis of 267 patients, Urban et al. used an ultrasound-guided approach to target the infrapatellar branches of the saphenous nerve, as well as branches of the femoral cutaneous nerve, for cryoanalgesia prior to a primary TKA [16]. The cryoanalgesia group required fewer opioids postoperatively up to 6 weeks after and had lower pain scores and lower length of stay. In a randomized sham-controlled pilot study of 16 subjects undergoing TKA, preoperative ultrasound-guided cryoneurolysis of the IBSN was performed. No statistics were performed due to the small sample size, but the findings suggested slight improvements in pain scores and opioid consumption up to 21 days after surgery [12]. More high quality clinical trials are needed to demonstrate the efficacy of ultrasound-guided cryoneurolysis of the IBSN and AFCN for postoperative analgesia following TKA.

#### Limb Amputations

For lower extremity amputations – such as below-the-knee amputation (BKA) and above-the-knee amputations (AKA) – reported nerve targets for ultrasound-guided cryoneurolysis were sciatic and femoral nerves (usually at distal components) [40, 46]. There are limited randomized controlled trials testing the efficacy of postoperative analgesia with cryoneurolysis and lower extremity amputations during the perioperative period, however, it has been described in a case series [40] and pilot study of 7 subjects [46]. The outcome of these studies suggest that perioperative cryoneurolysis of the femoral and sciatic nerves may reduce long-term postoperative pain and opioid use after limb amputation. On the other hand, the efficacy of cryoneurolysis for chronic phantom limb pain after amputation is further described in the chronic pain section of this review.

#### Mastectomies

Ultrasound-guided cryoneurolysis of the intercostal nerves has been used to pre-emptively treat postoperative pain following mastectomy [11, 41]. In a case series of three patients, it was reported patients were pain-free and did not require any supplemental opioid analgesics during the entire postoperative follow-up period [41]. A subsequent single-institution randomized sham-controlled clinical trial was performed on 60 subjects undergoing mastectomy. Subjects received either active or sham cryoneurolysis of the T2-T6 intercostal nerves in addition to paravertebral blocks (per standard care). On postoperative day 2, subjects who received active cryoneurolysis had statistically significant pain score reduction (0 versus 3). Furthermore, there was evidence for superior analgesia through month 12. In fact, during the first 3 weeks, cryoneurolysis lowered opioid use by 98%. At one year follow-up, chronic pain developed in 3% versus 17% of active and sham subjects, respectively [11]. This demonstrates the promising results from applying preoperative ultrasound-guided cryoneurolysis for patients undergoing mastectomy.

#### Burn and Trauma

Examples of cases reported to use cryoneurolysis in the burn/trauma environment include pain management for rib fractures [38, 39, 44] and skin grafting for burn patients [10, 36]. For rib fractures, all studies have been limited to case series, in which all demonstrated positive outcomes using ultrasound-guided cryoneurolysis for acute rib fracture pain [38, 39, 44]. For skin grafting of the anterior lateral thigh for burn patients, ultrasound-guided cryoneurolysis of the lateral femoral cutaneous nerve was performed [10, 36]. In a randomized controlled pilot study of 12 patients, those receiving active cryoneurolysis reported lower average and maximum pain scores and less opioids up to 7 days after the procedure [10].

#### Other Reports Related to Acute pain Management

There have also been several other case reports describing the use of cryoneurolysis for acute surgical pain indications, including intercostal cryoneurolysis for acute on chronic pain from breast ulcerations [37], intercostal cryoneurolysis for postoperative pain following percutaneous nephrolithotomy [42], and cryoneurolysis of the suprascapular nerve for rotator cuff surgery [43].

Most studies on ultrasound-guided or landmark-based cryoneurolysis for acute pain indications were limited to case reports or retrospective studies. However, the reported case series provide ongoing insights into the potential of this modality for treating acute pain for a period that can outlast more traditional approaches to acute pain management (e.g., peripheral regional anesthesia, neuraxial blocks, etc.). However, widespread adoption of this technology in the perioperative space will require appropriate billing/reimbursement workflows, equipment availability, and expertise training [55].

### Applications for Chronic Pain Management

Ultrasound-guided cryoneurolysis for chronic pain conditions has been described for several pain etiologies, including, but not limited to, chronic knee pain [6, 7, 33], chronic shoulder pain [25], painful neuropathies [18, 19, 30], post-mastectomy and -thoracotomy pain syndrome [29, 31], phantom limb pain following amputations [8, 21], facial pain/headaches [5, 9, 20], sacroiliac joint pain [22, 34], foot/ankle pain [17, 24], arm/hand pain [25, 27, 35], inguinal pain [26], meralgia paresthetica [28], and complex regional pain syndrome [23].

#### Chronic Knee Pain

Cryoneurolysis for treating pain from knee osteoarthritis has been previously described in a case series [33] and one RCT [7]. The IBSN is a target nerve for this condition. In a case series, peripheral nerve stimulation to identify the IBSN was used to guide cryoneurolysis treatment of non-surgical anterior knee pain [33]. In an RCT, 180 subjects with painful knee osteoarthritis were randomized to active versus sham cryoneurolysis of the IBSN to measure efficacy of improving the Western Ontario and McMaster Osteoarthritis Index (WOMAC) score over 30 days after intervention and pain [6]. At 1, 2, and 3 months after cryoneurolysis, the WOMAC scores were shown to improve in the cryoneurolysis cohort without any differences in serious or unanticipated adverse device events [7]. More studies are needed to assess the efficacy in other painful knee etiologies such as post-surgical pain as well as if croyneurolysis was used to treat other nerves of the knee, including the genicular nerves (e.g., superomedial, inferomedial, superolateral, and inferolateral genicular nerves) and AFCN.

#### Chronic Chest Wall Pain

Ultrasound-guided cryoneurolysis of the intercostal nerves is a potential intervention for pain in the truncal region. Two examples come from case reports demonstrating positive outcomes for a patient with post-mastectomy pain syndrome [31] and post-thoracotomy pain syndrome [29]. Repeated use of intercostal cryoneurolysis approximately every 6 months for 2.5 years was described to maintain analgesia in a patient with chronic post-mastectomy pain without any neurologic sequelae [31]. This demonstrates the potential use of cryoneurolysis for long-term management of chronic pain without causing nerve injury from repeated interventions. Analgesia from cryoneurolysis lasted around 4–6 months each time. Similar results were seen in a patient with chronic post-thoracotomy pain syndrome, in which intercostal cryoneurolysis provided 75% analgesia for 6 weeks followed by 50% pain relief for an additional 8 weeks [29]. These published reports highlight the potential of intercostal cryoneurolysis as a radiation-free, safe, and efficacious therapy for chronic post-surgical truncal pain; however, more high quality studies are needed to demonstrate the efficacy and duration of analgesia from this intervention.

### Facial Pain

Cryoneurolysis (either via landmark or ultrasound-guided approaches) of the occipital nerves is an option for managing pain from occipital neuralgia and cervicogenic headaches [5, 9, 20]. Cryoneurolysis of the occipital nerves has been reported in one RCT [5], a prospective non-randomized trial [9], and a retrospective observational study [20]. An RCT compared cryoneurolysis versus corticosteroid/local anesthetic treatment of the occipital nerves for treatment of cervicogenic headaches among 52 subjects [5]. In this study, both cryoneurolysis and corticosteroid treatment of the occipital nerves provided significant but temporary analgesia; however, statistically significant differences were not found. However, this may suggest larger scaled RCTs with adequate power are needed to demonstrate its efficacy in this population. In contrast, in a non-randomized multi-center pilot study (*n* = 26), cryoneurolysis of the occipital nerves was demonstrated to provide significant pain relief from occipital neuralgia 30 days after treatment with an acceptable safety profile [9]. Similarly, in a retrospective study of 38 patients, cryoneurolysis of the occipital nerves demonstrated some association with long-term pain relief [20]. In this study, patients with greater than or equal to 50% relief and less than 2 week duration of relief were treated with cryoneurolysis of the occipital nerves. Significance in duration and degree of analgesia was seen in women with at least 75% benefit with local anesthetic injections. These studies provided important data for potential efficacy of cryoneurolysis of the occipital nerves; however, highlight the need for higher quality RCTs that are adequately powered to clinically important outcomes to demonstrate its efficacy.

#### Neuropathies

Cryoneurolysis for painful peripheral neuropathies has also been described in the literature [18, 19, 30]. In a case series of patients with painful diabetic neuropathy of the foot, ultrasound-guided treatment of the superficial peroneal nerve (at the ankle) – performed after diagnostic blocks with lidocaine – demonstrated long-term improvement in pain in 2 out of the 3 subjects [30]. Patients were described to have not only significant analgesia, but subsequent improvement in ambulation. In a retrospective analysis of 24 patients, results from ultrasound-guided cryoneurolysis among patients with peripheral mononeuropathies were reported [19]. At one month after the procedure, a little over of half of the patients reported pain reduction of at least 30%. The reduction at 3 and 6 months was approximately 14% and 9%, respectively. In a prospective study of 22 patients, ultrasound-guided cryoneurolysis was performed for patients with refractory peripheral neuropathic pain [18]. Average pain levels were 8.3 ± 1.9 before intervention and 2.3 ± 2.5 at 1 month, 3.2 ± 2.5 at 3 months, 4.7 ± 2.7 at 6 months, and 5.1 ± 3.7 at 12 months afterward. These studies demonstrated the potential benefits of cryoneurolysis for chronic neuropathic pain; however, larger-scale RCTs are needed to definitively assess its efficacy for this patient population.

#### Sacroiliac Joint Pain

The sacroiliac joint is a common source of pain and discomfort for patients with lower back pain. Ultrasound-guided cryoneurolysis for sacroiliac pain has also been described as a case series [34] and retrospective observational analyses [22]. In the retrospective cohort analysis, 39 and 44 patients who received either cryoneurolysis or steroid injection, respectively, for sacroiliac joint pain were compared [22]. Both cohorts demonstrated significantly decreased pain scores from prior to procedure to immediately, 1 month, 3 months and 6 months post intervention. However, the pain scores were lower in the cryoneurolysis cohorts. Furthermore, more patients had ≥ 50% decrease in pain scores from baseline at all time points in the cryoneurolysis group. This study demonstrates the benefit of cryoneurolysis for sacroiliac joint pain compared to steroid injections; however an RCT is required to truly assess the difference in efficacy.

#### Phantom Limb Pain

One multi-center randomized sham-controlled, patient-blinded, crossover clinical trial was performed to assess the efficacy of ultrasound-guided cryoneurolysis of the sciatic and femoral nerves for treatment of chronic phantom limb pain following below-the-knee and above-the-knee amputation [8]. The primary outcome was average pain over the previous 72 h at 4 months after initial treatment. The study enrolled 144 patients from 6 institutions. While no difference was found in the primary outcome between sham and cryoneurolysis, there are several considerations that need to be considered: (1) the intervention protocol may not have been optimized for this specific population (i.e., perhaps longer duration of freeze cycles was required for these larger nerves as the study was limited to 2 freeze-thaw cycles of 2 min per nerve; and (2) heterogeneity of study population, in that it studied both below-the-knee and above-the-knee amputees – both which may have very different nerve distributions. For example, only the sciatic and femoral nerves were treated for the above-the-knee group, but this leaves out other potential nerves affected, including the obturator, posterior femoral cutaneous, and lateral femoral cutaneous nerves. Furthermore, there may be benefits in analgesia not measured prior to the 4 month study period. Thus, future studies are required with a more optimized intervention protocol with a more homogenous study population to definitively assess the efficacy of cryoneurolysis for lower extremity phantom limb pain treatment. For example, a current pilot RCT study is underway using longer freeze cycles of sciatic, femoral, obturator, and lateral femoral cutaneous nerves specifically for patients with above-the-knee amputations and phantom limb pain (clinicaltrials.gov, NCT06071715).

#### Other Chronic Pain in Extremities

The suprascapular nerve is a potential target for crynoeurolysis for chronic pain in the shoulder. In a case report, bilateral suprascapular nerve (at the suprascapular notch) cryoneurolysis was described for pain associated with glenohumeral osteoarthritis, in which the analgesic effects were apparent at 170 days after the procedure [25]. In another case report, a patient with multiple sclerosis received bilateral superficial fibular nerve cryoneurolysis to treat dorsal foot pain, in which the analgesic effects lasted 5.5 months before symptoms returned [24].

### Summary/Conclusion

Interventions such as percutaneous cryoanalgesia offer opportunities to improve patient postoperative recovery and chronic pain. There are still many questions that need to be answered. The number of necessary cryoanalgesia applications for each target, the duration of cryoanalgesia, the duration of thawing, and the specific design of the cryoprobe and cannula have yet to be standardized. One study has suggested a relationship between the duration of cryoanalgesia and the duration of overall anesthetic effect, indicating that there may be an ability to control the length of treatment effect.^19^ This finding has to be considered, however, along with the risks of partial ablations and development of hyperalgesia, allodynia, and severe dysesthesia.^39^ For both acute and chronic pain indications, more high quality RCTs are needed to definitively assess the efficacy of cryoneurolysis versus other standard therapies for a multitude of pain conditions.

## Data Availability

No datasets were generated or analysed during the current study.
